# Sex-Specific Effects of High Fat Diet on Indices of Metabolic Syndrome in 3xTg-AD Mice: Implications for Alzheimer's Disease

**DOI:** 10.1371/journal.pone.0078554

**Published:** 2013-10-28

**Authors:** Anna M. Barron, Emily R. Rosario, Reem Elteriefi, Christian J. Pike

**Affiliations:** Davis School of Gerontology, University of Southern California, Los Angeles, California, United States of America; Nathan Kline Institute and New York University School of Medicine, United States of America

## Abstract

Multiple factors of metabolic syndrome have been implicated in the pathogenesis of Alzheimer's disease (AD), including abdominal obesity, insulin resistance, endocrine dysfunction and dyslipidemia. High fat diet, a common experimental model of obesity and metabolic syndrome, has been shown to accelerate cognitive decline and AD-related neuropathology in animal models. However, sex interacts with the metabolic outcomes of high fat diet and, therefore, may alter neuropathological consequences of dietary manipulations. This study examines the effects of sex and high fat diet on metabolic and AD-related neuropathological outcomes in 3xTg-AD mice. Three month-old male and female 3xTg-AD mice were fed either standard or high fat diets for 4 months. Obesity was observed in all high fat fed mice; however, ectopic fat accumulation, hyperglycemia and hyperinsulinemia were observed only in males. Interestingly, despite the different metabolic outcomes of high fat diet, the neuropathological consequences were similar: both male and female mice maintained under high fat diet exhibited significant worsening in behavioral performance and hippocampal accumulation of β-amyloid protein. Because high fat diet resulted in obesity and increased AD-like pathology in both sexes, these data support a role of obesity-related factors in promoting AD pathogenesis.

## Introduction

Growing evidence suggests that metabolic syndrome may increase risk of cognitive decline and Alzheimer's disease (AD) [1]. Metabolic syndrome is a cluster of metabolic abnormalities including obesity, visceral adiposity, hyperinsulinemia, hyperglycemia, hypertension and hypercholesterolemia [2]; and is a leading health issue facing western societies owing to the high sucrose, high saturated fat content of the western diet [3]. Western diet and resulting metabolic abnormalities may promote AD-related neuropathology, with studies demonstrating that high fat diet in animal models of AD is associated with increased accumulation of the toxic β-amyloid peptide (Aβ) and impaired behavior [4]–[7]. However, sex has important influences on neural function and disease [8], as well as responses to metabolic stressors [9]. Sex differences in response to metabolic stressors may translate into differences in the effects of the western diet on AD neuropathology and may prove to be important for understanding mechanistic interactions between metabolic syndrome and AD neuropathology.

Sex differences in obesity, adiposity and insulin resistance are observed in both experimental animal models of metabolic syndrome [10] and humans [11]. In fact, in many rodent models, insulin resistance occurs rarely in females or exclusively in males [10]. Sex steroid hormones are believed to underlie sex differences in metabolic outcomes in response to stressors such as the western diet, with estrogens theorized to protect women until menopause [12]. Supporting this position, the prevalence of metabolic syndrome is higher in men than in similarly aged pre-menopausal women [11] and a higher level of adiposity is required in women to elicit metabolic disturbances [13]. Furthermore, the cognitive consequences of obesity may be more severe for men than women. For example, a recent study demonstrated that high fat diet impaired learning and memory and synaptic plasticity in obese male but not obese female mice [14]. In this study, more severe metabolic impairments were observed in the obese male than obese female mice, suggesting males may be more vulnerable than females to the impact of obesity on both metabolic homeostasis and deficits of learning and memory [14]. Similarly, in humans, obesity can exert more profound impairments in cognitive function in men in comparison to women [15]. Despite sex-specific metabolic phenotypes resulting from metabolic disturbances such as high fat diet, the effect of sex on neuropathological outcomes following high-fat feeding in animal models of AD has not been previously considered, and in some cases male and female mice have been combined in study groups [5], [6].

The current study examines the interactions between sex, metabolic indices and AD-related neuropathological and functional outcomes resulting from high fat diet in a transgenic mouse model of AD. Examining sex-dependent differences in vulnerability to obesity-induced insulin resistance provides new insights into the contributions of obesity to AD pathogenesis in the absence of other metabolic abnormalities.

## Materials and Methods

### Animals and surgical procedures

Male and female homozygous 3xTg-AD mice (APPswe, PS1M146V, tauP301L) [16] were bred and maintained at the University of Southern California vivarium facilities with food and water available *ad libitum*. Beginning at 3 months of age, mice were randomly assigned to groups (*n* = 6–8/group) maintained on their standard diet (14% kCal/fat; #8604 Harlan Teklad, Indianapolis, IN) or switched for a period of 4 months to a high calorie, high fat-diet (60%kCal/fat; TD.06414 Harlan Teklad). Comparison of the standard and high fat diet composition is shown in Table 1. Body weights were monitored weekly. All experimentation was carried out in strict accordance with the recommendations in the Guide for the Care and Use of Laboratory Animals of the National Institutes of Health and was approved by the Institutional Animal Care and Use Committee of the University of Southern California (Protocol No. 11492).

**Table 1:** Comparison of dietary fat and energy composition between the standard and high fat diets.

	Standard Diet	High Fat Diet
***Diet Components***		
% Soybean oil	∼1.5	3
% Lard	0	31
% Sucrose	0	12.1
***Diet Content***		
Energy density	3 kCal/g	5.1 kCal/g
Energy from fat	14%	60%
Cholesterol	50 mg/kg	300 mg/kg
***Fatty acid (% Total fat)***		
SFA	0.9	37
SFA	1.1	47
PUFA	2.1	16

SFA: Saturated fatty acids; MUFA: monounsaturated fatty acids, PUFA: polyunsaturated fatty acids.

### Tissue collection and preparation

Mice were fasted for 12 hours prior to sacrifice then deeply anesthetized (50 mg sodium pentobarbital/kg body weight, i.p.). Blood was collected via cardiac puncture and fasting blood glucose assessed. Serum was collected from the remaining blood and stored at −80°C. Mice were then intracardially perfused with ice-cold, sterile 0.1 M PBS (pH 7.4) and the brain was submersion fixed in freshly prepared 4% paraformaldehyde/0.1 M PBS (pH 7.4) for immunohistochemistry. Retroperitoneal fat pads were collected at necropsy and weighed as an indicator of visceral adiposity and body composition.

### Liver histopathology

Fixed liver was cryoprotected overnight in 30% sucrose then sectioned at 7 µm using a cryostat. Sections were stained with haematoxylin QS (Vector Laboratories, CA, USA) according to manufacturer's instructions, then dehydrated, cover-slipped with permanent mounting medium, and examined under light microscopy. Ectopic fat deposits dissolve during preparation of the specimen, resulting in white lobules at the sites where fat was located [17]. To quantify microvesicular and macrovesicular hepatic steatosis, the area and number of lipid droplets were measured using the particle sizing function in Image J, with macrovesicules defined as greater than 15 µm in diameter [18].

### Glucose, insulin and sex hormone measurements

Fasting blood glucose was assessed monthly. Blood glucose was assessed using a Precision Xtra Glucometer (Abbott Laboratories, IL, USA) from whole blood collected via the tail vein while the mouse was under isofluorane general anesthesia. Serum insulin levels were assessed at completion of the experiment using a rodent insulin ELISA (Millipore, MA, USA) according to the manufacturer's instructions. Insulin resistance was assessed with the homeostasis model (HOMA-IR), calculated as: (fasting blood glucose (mg/dL) × fasting insulin (mU/L))/405. Serum levels of testosterone (Diagnostic System Laboratories, Texas, US) in male samples and estradiol (Calbiochem, MA, USA) in female samples were assessed by ELISA kit according to manufacturer's instructions. An outlier was excluded from statistical analyses in the high fat fed female group (>3 standard deviations from mean).

### Immunohistochemistry

Fixed hemibrains were exhaustively sectioned in the horizontal plane at 40 µM using a vibrating microtome. Every eighth section was pretreated with formic acid (99%) for 5 minutes, then immunolabeled with antibodies directed against Aβ (#71-58000, 1∶300 dilution, Zymed, CA, USA) or a phospho-specific epitope of tau (AT8, 1∶1000 dilution, Peirce, IL, USA). Immunoreactivity was visualized using ABC Vector Elite and DAB kits (Vector Laboratories, CA, USA) as previously described [19].

For quantification of Aβ load, grayscale images of high magnification fields (420×330 µm) were digitally captured (CCD camera coupled to Olympus Optical BX40 microscope) then thresholded at a predetermined, constant value using NIH Image 1.61 to create a binary image identifying positive and negative immunolabeling. Immunoreactive load was calculated as percentage of total pixel area positively labeled, as previously described [20]. Mean load values were averaged from two to three non-overlapping fields from each brain region in five sections per animal. AT8 immunoreactive neurons meeting the criteria of strong immunoreactivity over the entire cell surface were counted; numbers of AT8 positive neurons were summed across sections [21]. Experimenters were blinded to treatment condition during immunostaining and quantification procedures.

### Behavioral assessment

Spontaneous alternation behavior (SAB), a hippocampal-dependent measure of working memory and attention [22], was assessed in the Y-maze 24 hours prior to sacrifice. We have reliably observed a strong correlation between Aβ neuropathology and Y-maze SAB impairment in both male and female 3xTg-AD mice [19]. Mice were allowed to explore the Y-maze freely for 5 minutes or until 15 arm choices were made. SAB score was calculated as the number of alternations divided by the total number of alternation opportunities as previously described [19]. The first 15 arm choices were used to calculate the SAB score to avoid confounding effects of differences in exploratory activity between treatment groups.

To assess object recognition, mice were placed in the center of an open field maze (60 cm × 30 cm) and allowed to freely explore for 6 minutes. Following a 1 h interval, mice were again placed in the center of the maze between 2 identical objects and allowed to freely explore for 3 minutes. Frequencies of left and right object explorations were scored to test for spatial bias. After a 2 h inter-trial interval, one object was replaced (novel object) and the mouse allowed to freely explore for 3 minutes (2 hr Probe trial). This was repeated again after an 18 hr interval (18 hr Probe trial). The maze and objects were thoroughly cleaned with ethanol between trials. Frequency of novel (F_n_) and familiar (F_f_) object explorations were scored. The discrimination index was calculated by subtracting the frequency of the familiar object explorations from the frequency of novel object explorations (F_n_ − F_f_) divided by the total number of object explorations (F_n_ + F_f_).

### Statistical analyses

Data was analyzed by two-way ANOVA using the Statistical Package for Social Sciences (SPSS: version 11.5; SPSS Inc., IL, USA). Glucose and body weights data were analyzed by repeated measures two-way ANOVA. Pair-wise comparisons were made for significant interactions. All data are presented as mean ± SEM. Significance was set at a threshold of *p*<0.05.

## Results

### Obesity and adiposity

**Figure 1:**
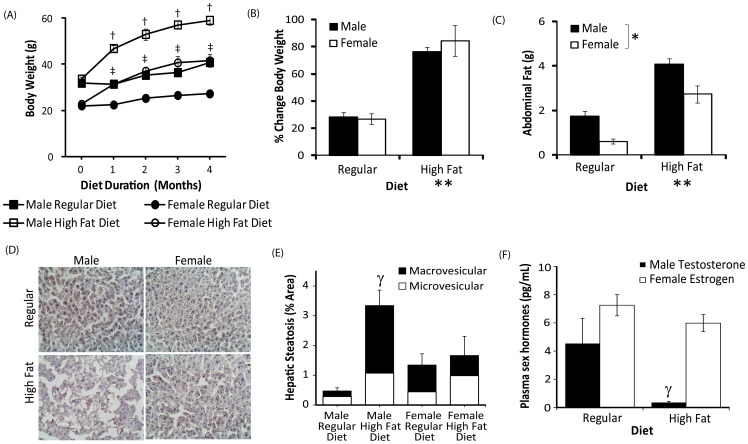
Obesity and adiposity in male and female high fat fed 3xTg-AD mice. A, Body weight in male and female 3xTg-AD mice across the 4 month feeding period. B, Percent change in body weight relative to baseline weight after 4 months of feeding with either standard or high fat diet. C, Abdominal retroperitoneal fat pad weight in male (solid bars) and female (open bars) 3xTg-AD mice. D, Representative images of hematoxylin-stained livers in male and female mice fed regular and high fat diets. E, Quantification of microvesicular (<15 µm) and macrovesicular (>15 µm) fat accumulation in liver. F. Serum testosterone and estradiol levels in male and female mice respectively. Data presented as mean ± SEM.† *p*<0.001 relative to matched time point males fed regular diet. ‡*p*<0.001 relative to matched time point females with regular diet. **p<0.001 relative to regular diet fed mice. *p<0.01 male versus female mice. γ*p*<0.05 relative to sex-matched regular diet fed mice.

Following the 4-month treatment period, male mice had significantly greater body weights compared to female mice (Fig. 1A), however no effect of sex was observed on the percent change in body weight over the duration of the study (Fig. 1B). Male and female mice fed the standard diet increased body weight by more than 25% due to normal growth over the duration of the study. High fat diet induced robust weight gain in both male and female mice. Following the first month of exposure to high fat diet, both male and female mice 3xTg-AD mice increased body weight by more than 40%. By the completion of the study, the high fat fed mice had increased from baseline body weight by >75%.

Increased body weight in the high fat fed mice reflected an increase in visceral adiposity, with significantly increased abdominal fat weights observed in high fat fed mice (*F* = 98.9, *p*<0.001; Fig. 1C). Larger abdominal fat depots were observed in male compared to female mice irrespective of diet (*F* = 90.3, *p*<0.001). However, despite larger gross abdominal fat pads in male mice, abdominal fat represented ∼7% of total body mass in male and ∼6% of total body mass in female high fat fed mice, indicating similar visceral adiposity composition in the male and female mice.

Despite similar levels of obesity and visceral adiposity composition in high fat fed male and female mice, histological examination of the liver revealed significantly increased ectopic fat accumulation in the livers of high fat fed male mice compared to all other groups (*F* = 6.89, *p*<0.05), while high fat diet did not significantly alter micro- and macrovesicular fat accumulation in the liver of female mice (Fig. 1D, 1E; *p* = 0.59).

### Obesity induced hypogonadism in male mice

Serum testosterone levels were markedly reduced in high fat fed male mice compared to male mice fed the regular diet (Fig. 1F; *t* = 6.21, *p*<0.001) indicating hypogonadism. No difference was observed in estradiol levels between regular and high fat female mice (Fig. 1F; *t* = 0.95, *p* = 0.7).

### Glucose metabolism

**Figure 2:**
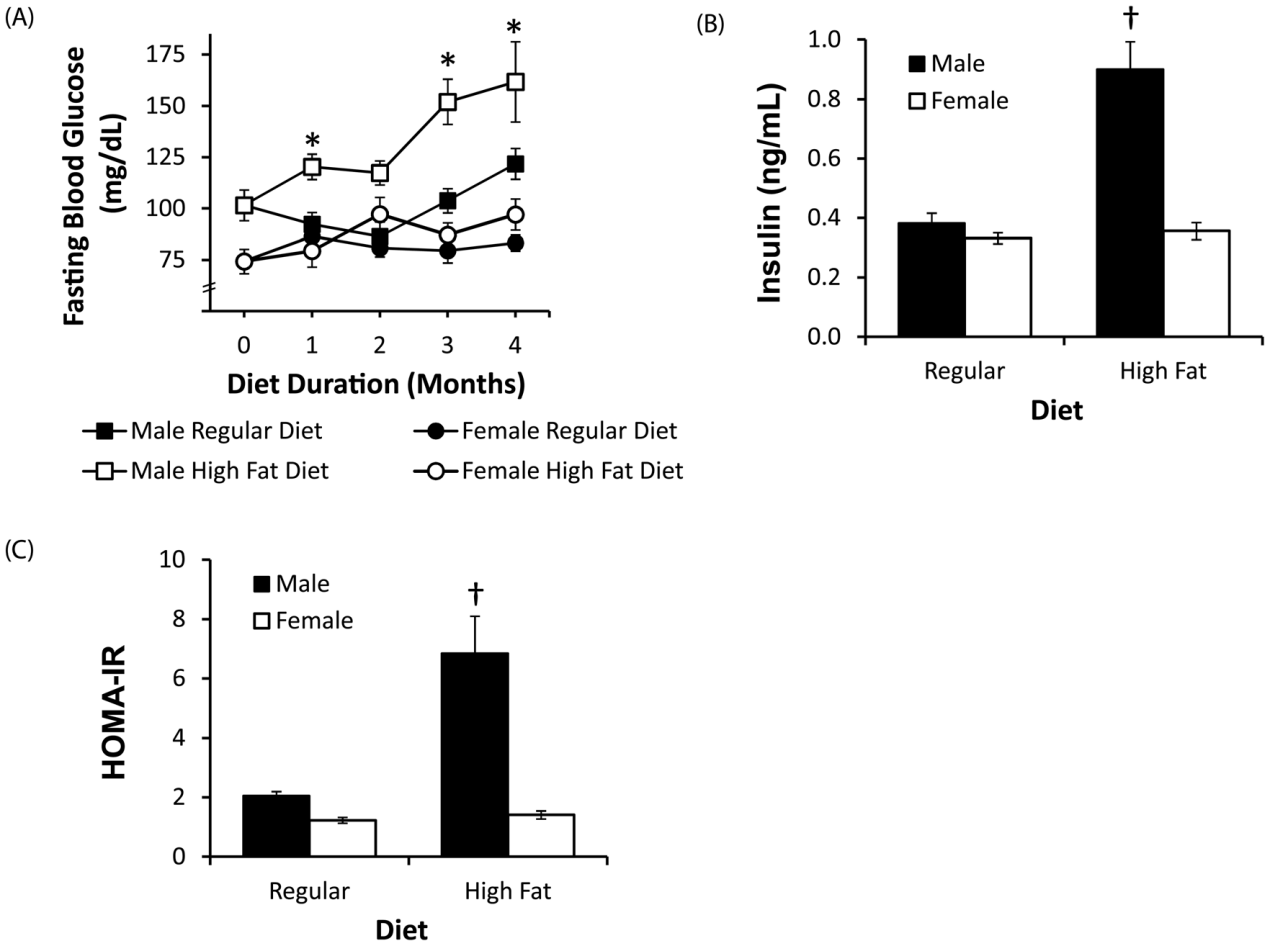
Elevated fasting blood glucose and insulin levels in high fat fed male but not female 3xTg-AD mice. A, Fasting blood glucose concentrations in male and female 3xTg-AD mice across the 4 month feeding period. B, Fasting insulin concentrations in male (solid bars) and female (open bars) 3xTg-AD mice after 4 months of feeding with either standard or high fat diet. C, Insulin resistance index, HOMA-IR. Data presented as mean ± SEM. *p<0.001 relative to standard diet group at the matched sex and time point; † p<0.001 relative to matched sex standard diet group.

Analyses of metabolic changes showed that high fat diet promoted hyperglycemia and hyperinsulinemia in male but not female 3xTg-AD mice, resulting in a significant interaction between sex and diet on fasting blood levels of glucose (*F* = 12.8, *p*<0.001) and insulin (*F* = 27.8, *p*<0.001). Significantly increased fasting blood glucose levels were observed in high fat fed male (*p*<0.001), but not female mice (*p* = 0.25), compared to sex-matched regular diet groups (Fig. 2A). Similarly, significantly increased insulin levels were observed in male (*p*<0.001), but not female (*p* = 0.70), 3xTg-AD mice after 4 months of high fat diet compared to sex-matched standard diet groups (Fig. 2B). These changes in glucose and insulin levels in the male high fat fed 3xTgAD mice reflected a significant increase in the HOMA-IR index, a measure of insulin resistance (*p*<0.001; Fig. 2C).

**Figure 3:**
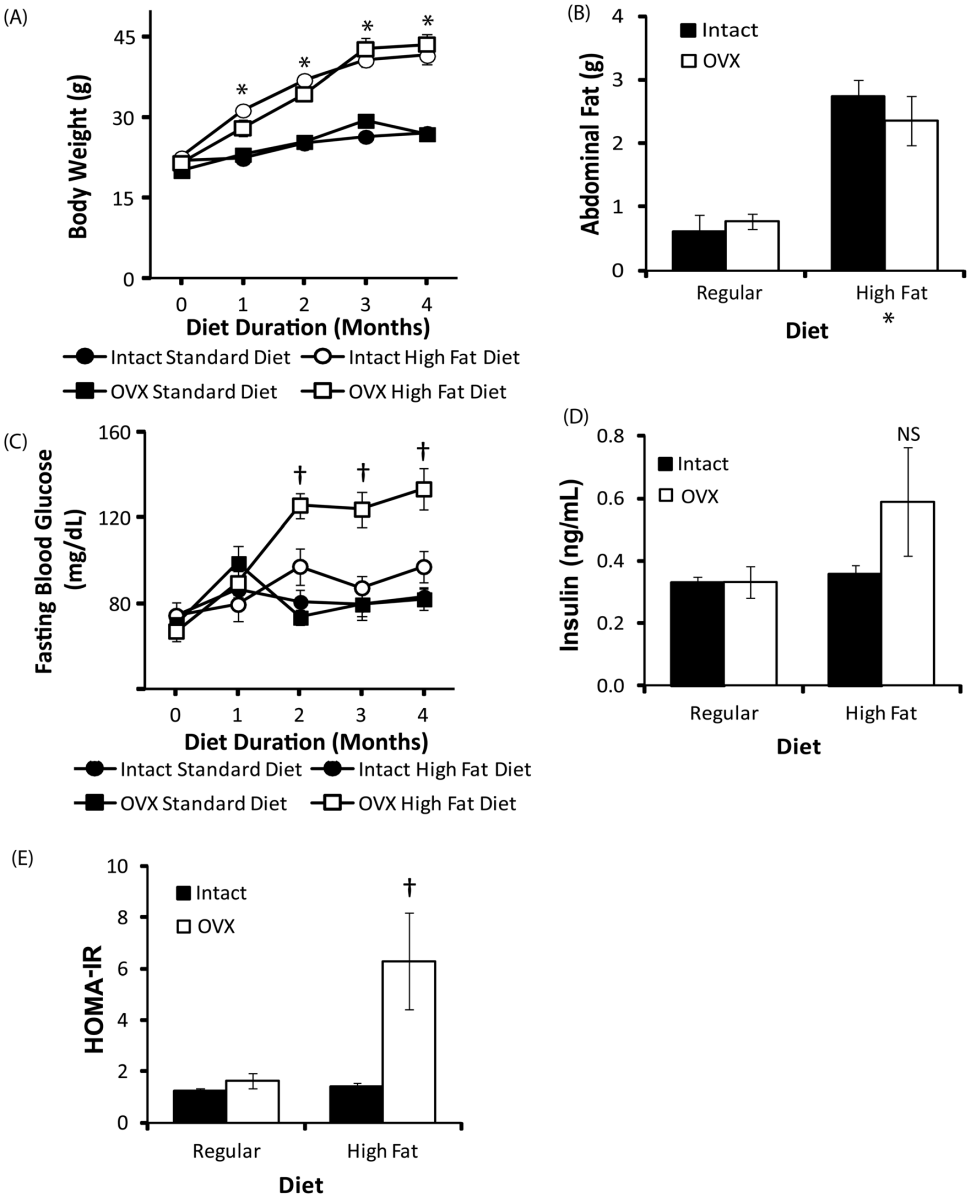
Ovarian hormones protect high fat fed female mice against hyperglycemia. A, Body weight in female 3xTg-AD mice was not affected by ovariectomy (OVX) in either standard or high fat groups. B, High fat diet increased abdominal retroperitoneal fat pad weight in sham-operated (solid bars) and ovariectomised (open bars) female 3xTg-AD mice equally. C, High fat diet increased fasting glucose concentrations in high fat fed female 3xTg-AD mice following ovariectomy D, Fasting insulin concentrations in sham-operated (solid bars) and ovariectomised (open bars) female 3xTg-AD mice after 4 months of feeding with either standard or high fat diet. C, Insulin resistance index, HOMA-IR. Data presented as mean ± SEM. *p<0.001 relative to regular diet fed mice; † *p*<0.001 relative to all other groups.

To investigate the potential protective role of the ovarian hormones in the lack of hyperinsulinemia observed in the high fat fed female mice, we compared the effect of high fat feeding in intact and ovariectomised (OVX) mice on body weight, adiposity and fasting glucose levels (Fig. 3). OVX did not significantly affect measures of body weight (*F* = 2.42, *p* = 0.13) or abdominal fat (*F* = 0.23, *p* = 0.63). A significant interaction between OVX and diet was observed on fasting blood glucose (*F* = 9.43, *p* = 0.005) and the HOMA-IR index (*F* = 5.65, *p* = 0.03). A trend towards elevated insulin levels in OVX high fat fed female approached statistical significance (*F* = 2.55, *p* = 0.08). Fasting blood glucose levels were significantly elevated in OVX mice after 2 months of high fat feeding compared to all other groups (*p*<0.001). The HOMA-IR index of insulin resistance was significantly elevated in high-fat fed OVX mice compared to all other groups (*p*<0.005).

### Behavior

**Figure 4:**
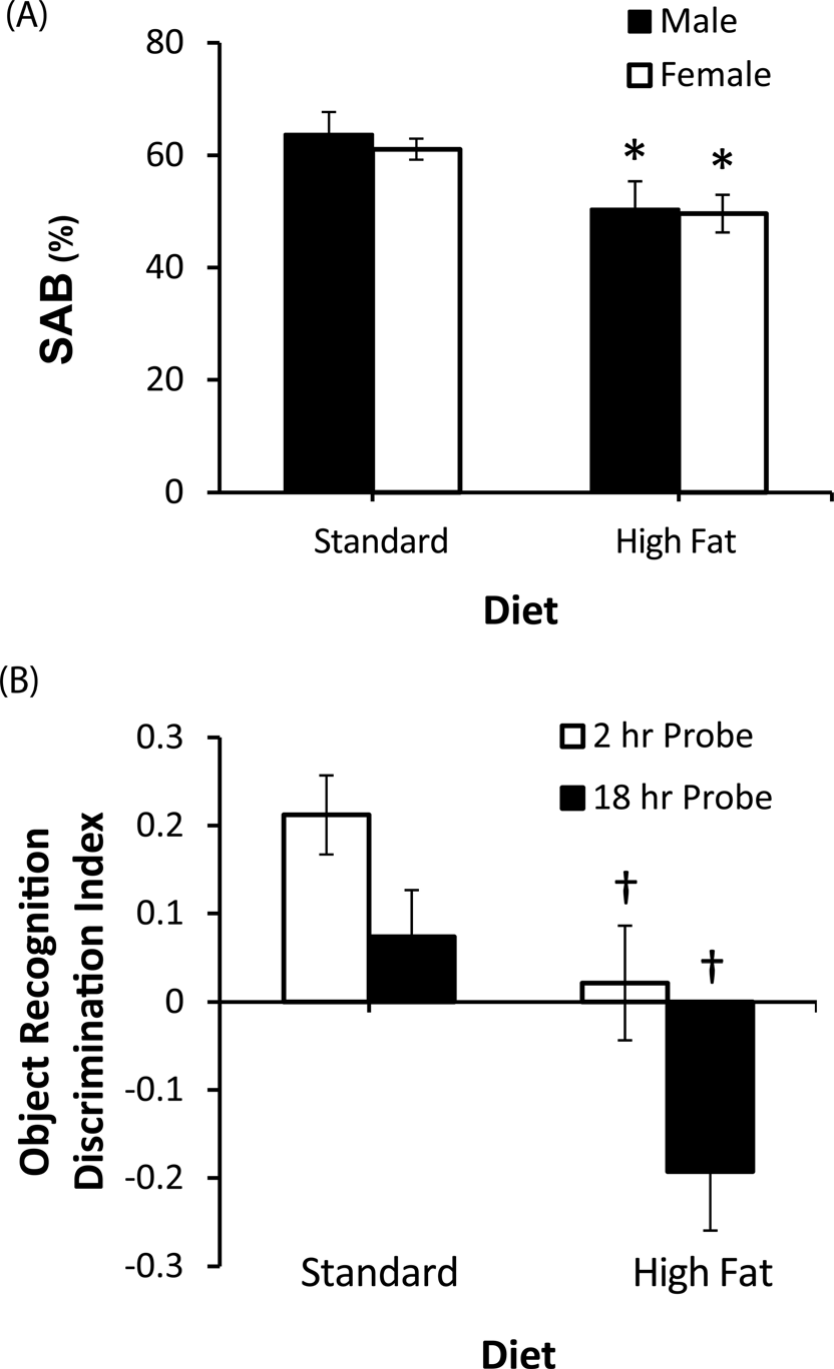
Impaired behavioral deficits in high fat fed 3xTg-AD mice. A, Spontaneous alternation behavior (SAB) in the Y-maze in standard and high fat fed male and female 3xTg-AD mice. B, Object recognition performance in standard and high fat fed female 3xTg-AD mice at 2 and 18 hrs after training (2 and 18 hr probe). *p<0.001 relative to matched sex with standard diet. Data presented as mean ± SEM. *p<0.001 relative to matched sex with standard diet. † *p*<0.001 relative to matched probe trial.

In the Y-maze task, decreased exploratory activity was observed in male mice fed the high fat compared to standard diet, with the high fat group making significantly fewer arm choices (*F* = 8.58, *p*<0.05). No significant difference in number of arm choices was observed between female mice fed the standard and high fat diets (*F* = 0.625, *p* = 0.54). To control for potential performance differences in spontaneous alternation behavior (SAB) in the Y-maze related to higher number of arm entries, only data from the first 15 arm choices was included for analysis of SAB performance across all groups. A significant effect of diet was observed on SAB performance (*F* = 12.18, *p* = 0.002), with impaired SAB observed in both male and female mice fed the high fat diet (Fig. 4A). No significant effect of sex was observed on SAB (*F* = 0.23, *p* = 0.64) and no interaction was observed between diet and sex (*F* = 0.06, *p* = 0.80). In the male 3xTg-AD mice, SAB performance showed significant negative correlations with glucose (r = −0.58, *p*<0.05) and insulin (r = −0.61, *p*<0.05) levels. In contrast, there were no significant correlations in female 3xTg-AD mice between SAB performance and either glucose (r = −0.14, *p* = 0.64) or insulin (r = 0.29, *p* = 0.93) levels.

Due to the marked decrease in exploratory activity in high fat fed male mice, quantification of object recognition performance was not possible in this group. In female mice, no significant difference in total exploratory activity in the open field maze was observed between standard (16±2.07 total object explorations) and high fat fed female mice (14±1.96 total object explorations). Impaired performance in novel object recognition was observed in female mice fed the high fat diet (*F* = 8.9, *p*<0.01), with a reduced discrimination index observed in the high fat fed mice at both the 2 hr and 18 hr probe trials (Fig. 4B).

### Aβ accumulation and tau hyperphophorylation

**Figure 5:**
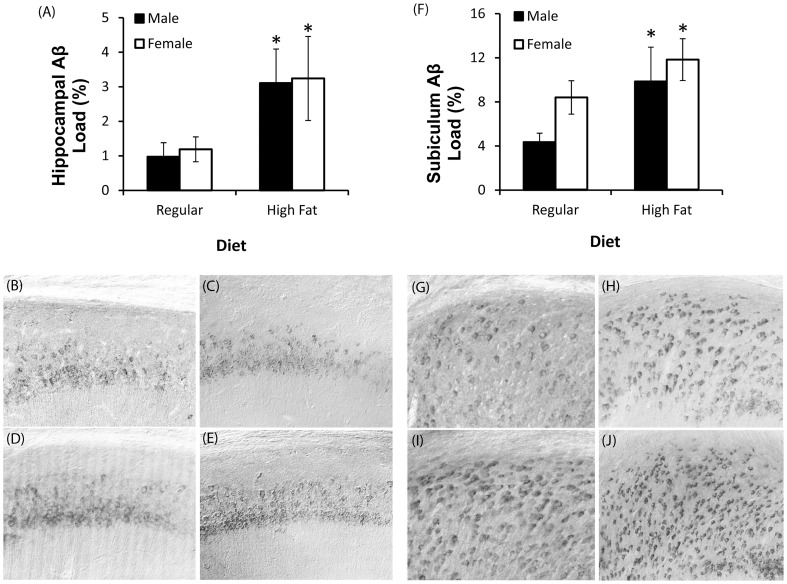
High fat diet increases Aβ accumulation in the hippocampus of male and female 3xTg-AD mice. A, Hippocampus CA1 Aβ immunoreactivity load values. B–E, Representative photomicrographs show Aβ immunoreactivity in the hippocampus CA1 regions in standard diet fed male (B) and female (C) 3xTg-AD mice, and high fat fed male (D) and female (E) 3xTg-AD mice. F, Subiculum Aβ immunoreactivity load values. G–J, Representative photomicrographs show Aβ immunoreactivity in the subiculum regions in standard diet fed male (G) and female (H) 3xTg-AD mice, and high fat fed male (I) and female (J) 3xTg-AD mice. Data presented as means ± SEM. *p<0.05 compared to all other groups. *p<0.001 relative to matched sex with standard diet.

Consistent with our previous findings [19], immunohistochemical Aβ load was more severe in the subiculum of female compared to male 3xTg-AD mice maintained on standard diet (*t* = −2.2, *p*<0.05). Significantly increased immunoreactive Aβ load was observed in both the hippocampus CA1 (*F* = 6.51, *p*<0.02; Figure 5A–E) and the subiculum (*F* = 5.04, *p*<0.05; Figure 5F–J) of high fat fed mice in both sexes. Despite sex specific effects of the high fat diet on metabolic measures, no significant interaction between sex and diet were observed on either hippocampal CA1 (*F* = 0.01, *p* = 0.96) or subiculum Aβ loads (*F* = 0.27, *p* = 0.61).

No significant effect of diet was observed on AT8 immunoreactivity in the female mice (Standard diet  =  9.0±2.44; HF diet  =  7.6±2.58). In 7 month-old male 3xTgAD mice, insufficient AT8 immunoreactivity is observed for quantitative analysis.

## Discussion

The high sucrose, high saturated fat content typical of the western diet has led to increasing rates of obesity and metabolic syndrome, and recent studies indicate that these lifestyle-related diseases may also be an important factor in the development of AD [23], [24]. However, it remains unclear what facets of metabolic syndrome are mechanistically implicated in AD pathogenesis. Comorbidity of obesity, insulin resistance, dyslipidemia, endocrine dysfunction, inflammation and leptin resistance in diet-induced models of metabolic syndrome, which emulates the most common cause of metabolic syndrome in human populations, makes it difficult to dissociate factors contributing to AD risk. In the current study, ovarian hormones protected female mice against insulin resistance, resulting in a phenotype described in humans as metabolically benign obesity, a phenomenon observed in over 25% of obese individuals where increases in visceral and total body fat are not accompanied by insulin resistance [25], [26]. Although we found the obese phenotype in 3xTg-AD female mice to be metabolically benign, it was as detrimental on measures of AD-related pathology as the metabolically dysfunctional phenotype observed in male 3xTg-AD mice. These findings suggest that obesity-related factors, even prior to the development of insulin resistance, may play an important role in increasing risk of AD in metabolic syndrome.

Our findings are consistent with previous studies which have also demonstrated that high fat diet promotes Aβ accumulation and impairs cognitive performance in 3xTg-AD [5] and Tg2576 mice [4], [6]. While these previous findings and studies in non-obese models of diabetes [27]–[31] provide strong evidence that impaired insulin signaling plays a key role in Aβ accumulation and AD pathogenesis, our findings indicate that obesity, even prior to the development of insulin resistance, may also play an important role in susceptibility to AD.

Recent studies have implicated adipose tissue in the generation of Aβ, finding that APP, the parent protein of Aβ, is highly expressed in adipose tissue, and that adipose APP expression is upregulated in obese individuals [32]. Further, plasma Aβ levels correlate with adipocyte APP expression in obese individuals [33]. These findings suggest that the adipose tissue may directly promote increased Aβ production. Chronic systemic inflammation resulting from free fatty acid and cytokine production by adipose tissue may also contribute to susceptibility to AD neuropathology. High fat feeding has been previously found to promote neuroinflammation, increasing neurocortical levels of glial fibrillary acidic protein, a marker of astrocyte activation, in both wild type and 3xTg-AD mice [5]. Inflammation is a key pathological feature of AD and gliosis can promote Aβ burden [34]. Increased Aβ production and inflammatory factors produced by adipose tissue may have contributed to acceleration of Aβ neuropathology observed in the high fat fed 3xTg-AD mice in the current study. Furthermore, the contribution of obesity to AD-related pathogenesis is an important consideration in interpretation of findings using experimental models of type II diabetes where obesity is a comorbid condition [4].

Our findings also indicate that metabolic phenotypes resulting from high fat feeding are dependent upon sex and, therefore, reproductive hormone status may be an important consideration for experimental design and interpretation when examining dietary effects on AD related neuropathology. The influence of sex and hormone status on neuropathological outcomes in diet-induced obesity models has not been previously considered and, in some cases, male and female mice have been combined in study groups [5], [6]. We found that high fat diet resulted in significant depletion of testosterone in males, but not significant loss of estrogens in females. Obesity-induced hypogonadism has been previously described in rats [35] and humans [36], [37], and evidence suggests that the loss of testosterone in males may in turn accelerate obesity and metabolic dysfunction [38]–[41]. In fact, clinical studies have found that testosterone therapy can reduce features of metabolic syndrome [42]–[45]. The interactions between testosterone, metabolic syndrome and AD-related neuropathology require further investigation and may present an opportunity for preventative intervention.

## Conclusions

Our findings support the notion that visceral obesity may accelerate Aβ burden independent of insulin-related dysfunction. Obesity encompasses a wide range of factors linked to AD pathogenesis, including adipose dysfunction, inflammatory, endocrine, lipid and vascular changes. Epidemiological studies indicate the relationship between obesity and AD is age-dependent, with obesity in mid-life associated with increased risk of AD in late life [46]–[52]. Importantly, this suggests that obesity-related factors may influence AD pathology in preclinical stages, representing the potential for preventative intervention.
